# Humic Acid Recovery from Leachate Nanofiltration Concentrate Using Halloysite Nanotube-Coated Tubular Ceramic Ultrafiltration Membrane

**DOI:** 10.3390/membranes16070236

**Published:** 2026-07-10

**Authors:** Sultan Akarçay Demir, Gamze Varank, Derya Y. Koseoglu-Imer, Gülay Arslan Cene, Emine Can-Güven, Senem Yazici Guvenc, Oruc Kaan Turk

**Affiliations:** 1 Department of Environmental Engineering, Faculty of Civil Engineering, Yildiz Technical University, 34220 Istanbul, Türkiye; sultan.demir1@std.yildiz.edu.tr (S.A.D.); ecguven@yildiz.edu.tr (E.C.-G.); syazici@yildiz.edu.tr (S.Y.G.); oturk@yildiz.edu.tr (O.K.T.); 2 Department of Environmental Engineering, Istanbul Technical University, 34000 Istanbul, Türkiye; imerd@itu.edu.tr; 3 Department of Environmental Engineering, Faculty of Engineering, Marmara University, 34584 Istanbul, Türkiye; gulay.arslan@marmara.edu.tr

**Keywords:** adsorption, desorption, halloysite nanotube, humic acid recovery, nanofiltration concentrate

## Abstract

Landfill wastewater is a serious environmental problem and represents a high-concentration source of valuable organic compounds such as humic acids (HAs). The nanofiltration (NF) concentrate generated during treatment poses an even more significant environmental challenge, and the recovery of these substances is compatible with circular economy principles but requires innovative, pollution-resistant separation technologies. This study presents a novel hybrid approach for HA recovery by integrating naturally occurring clay minerals, such as halloysite nanotubes (HNTs), as a dynamic coating layer onto tube-shaped ceramic ultrafiltration membranes. The research was conducted in two stages: batch adsorption–desorption experiments followed by membrane integration. In the first stage, the batch adsorption studies showed that HA adsorption by HNTs followed the Freundlich isotherm model. The maximum HA adsorption capacity for HNTs increased with increasing initial concentration. In desorption studies, recovery rates of 74.6% were achieved with 1.5 N sodium hydroxide (NaOH) and 67.5% with 1.5 N potassium hydroxide (KOH). In membrane studies, the optimum HNT coating concentration was determined as 0.05 g/L. While an average removal efficiency of 85.3% was obtained in synthetic HA filtration, the desorption efficiency after regeneration was around 35–37%. In experiments with real NF concentrate, HA removal efficiencies ranged from 19 to 64% for concentrations of 5, 10, and 20 mg/L, with the highest desorption efficiency (59.3%) obtained in the 10 mg/L NF concentrate. The results reveal that the complex structure and competing components in the real wastewater matrix limit the removal and recovery performance compared to synthetic solutions.

## 1. Introduction

Landfills are complex ecosystems where anaerobic and aerobic processes occur simultaneously, with organic matter being decomposed by microorganisms. During these processes, organic matter is converted into simpler compounds and insoluble organic molecules because of various biochemical reactions [1]. Humic substances (HSs) constitute a significant portion of these molecules. HSs are heterogeneous organic compounds in which organic carbon is stored [2]. HSs have properties that directly affect ecological balances such as the carbon cycle, water quality, and soil fertility [3]. These substances, which are of great importance in terms of increasing soil fertility and promoting plant growth, increase the water-holding capacity of soil while facilitating easier uptake of mineral nutrients by plants. In addition, the buffering capacity of HSs helps maintain the pH stability of soil and water [4]. These properties make it possible to use them as a natural fertilizer or soil conditioner in agricultural applications [5].

The formation of HSs in leachate depends on various factors such as the type of waste, landfill conditions, and biological decomposition processes. These substances generally have very complex structures and are found in leachate in the form of dissolved organic carbon (DOC) [6]. Various fractions such as humic acids (HAs), fulvic acids (FAs), and humin (HU) are formed during decomposition processes. The concentration of HSs in leachate can vary depending on factors such as the age of the landfill, the biodiversity of the waste, and the decomposition conditions.

The chemical structures of FAs, HAs, and HU are generally similar despite differences in some functional groups. However, in terms of agricultural applications, FAs and HAs stand out as the fractions that provide the highest benefit [7]. HSs have the potential to be used in various fields such as agriculture, environmental protection, and industrial applications. HSs recovered from wastewater or natural sources can be used as soil conditioners. These substances are known to increase the water-holding capacity of the soil, promote plant growth, and improve the microbial activity of the soil [8,9,10]. In addition, thanks to their carbon-rich structures, HSs can contribute to long-term carbon capture strategies by increasing the organic matter level in the soil. Therefore, the recovery of HSs as economically valuable products from treatment processes offers significant opportunities for both sustainable resource management and agricultural applications [3,8].

In landfills, leachate concentrates are formed as a result of membrane processes commonly used in leachate treatment. These concentrations contain complex organic structures where HSs are concentrated. Membrane technologies such as nanofiltration (NF) and reverse osmosis reduce (RO) the organic matter loading of leachate, while the majority of organic carbon accumulates in the concentrate phase. HS concentrations in the concentrate can generally reach much higher levels than in the raw leachate [11,12]. Studies in the literature show that HS concentrations in membrane concentrate streams can vary between 500 and 5000 mg/L [13,14,15]. These high concentrations necessitate the development of suitable technologies to reduce environmental impacts and potentially recover HSs.

Current management systems lack the technological infrastructure and regulatory framework for the recovery of such components [16]. The methods used in the recovery of HSs should be selected depending on the specific requirements of the process. Adsorption and membrane technologies stand out due to their low cost and wide application potential, while electrochemical and advanced oxidation methods are more effective removal mechanisms. Nanotechnology offers an innovative approach to the recovery of HSs. The use of nanoparticles as adsorbents can remove HSs faster and more effectively than traditional adsorbents due to their high surface area and functional surface groups [17]. Materials such as magnetic nanoparticles, metal oxide nanoparticles, and carbon nanotubes are particularly used. Magnetic nanoparticles can be easily recovered and reused after processing.

Halloysite nanotubes (HNTs) are a unique, naturally occurring, double-layered clay mineral with a nanotube structure composed of aluminum, silicon, hydrogen, and oxygen. Their natural formation allows them to exhibit diverse physical and chemical properties. The use of HNTs, particularly in the recovery of HSs like HAs, is noteworthy for sustainable water treatment and wastewater management. The literature has shown the effectiveness of HNTs as an adsorbent in the separation of similar organic substances such as HAs and FAs [18,19,20].

In recent years, modification methods using nanomaterials and tailored interfacial layers have been employed to improve the surface properties, selectivity, and operational stability of ceramic and hybrid membrane systems. In particular, clay-based nanostructures can be integrated into membrane surfaces to increase hydrophilicity and adsorption capacity. In addition to nanomaterial incorporation, recent studies have emphasized that the chemical configuration, interfacial compatibility, and structural stability of the modified layer play a critical role in maintaining membrane performance under harsh or specialized filtration conditions. For example, Xue et al. [21] developed a polyelectrolyte-assisted interfacial polymerization strategy in which a PEI/PSS interlayer regulated the distribution of reactive amine groups and promoted the formation of a uniform, defect-suppressed polyurea selective layer with high acid and alkali resistance [21]. This approach highlights that the performance of modified membrane systems depends not only on the intrinsic properties of the active material but also on the stability and homogeneity of the interfacial layer formed on the membrane surface. Hybrid systems combining HNTs and membrane technologies offer effective solutions for HS separation and recovery. In these systems, the adsorption capacity of HNTs is combined with the selective separation ability of membrane filtration. Various variations of this approach have been investigated in the literature. Eskitoros-Togay et al. produced composite membranes containing HNTs using the electrospinning method [22]. Ouda et al. combined magnetic nanocomposite halloysite materials with ultrafiltration (UF) membranes to achieve simultaneous recovery of HAs [19]. Al-Mutwalli et al. achieved a HA removal efficiency of 70.6% under optimum conditions in their experiments with nanoclay-coated ceramic membranes [23].

Although the literature indicates that integrating nanomaterials with membrane separation can enhance adsorption capacity and mitigate operational limitations during resource recovery, studies utilizing natural nanoclays such as HNTs for dynamic coating applications remain limited. The primary objective of this study was to systematically evaluate the performance of an HNT-coated tubular ceramic ultrafiltration membrane system designed specifically for the recovery of HAs. High-purity HAs was employed as the model target compound in the synthetic experiments. To achieve this objective, a comprehensive three-step methodological workflow was implemented: (i) baseline batch adsorption and desorption experiments were conducted to determine the thermodynamic and kinetic profiles of HA adsorption onto raw HNT powders; (ii) tubular ceramic membranes were dynamically coated under optimized crossflow conditions to establish a stable HNT layer; and (iii) dynamic filtration and chemical regeneration tests were sequentially performed using both controlled synthetic HA solutions and real leachate NF concentrate streams. A particular strength of this study is the evaluation of the proposed hybrid system using a real leachate NF concentrate, rather than relying solely on synthetic HA solutions. Through this structured approach, this work aims to demonstrate the practical viability and unique potential of HNT-modified hybrid systems for transforming real wastewater concentrates into sustainable material sources.

## 2. Materials and Methods

### 2.1. Materials and Chemicals

High-purity HAs (Sigma-Aldrich, St. Louis, MO, USA) in sodium salt form were used as the model contaminant in the experimental study. The HA stock solution (2000 mg/L) was prepared by dissolving the HA sodium salt in distilled water, covered to protect from light and heat, and stored at +4 °C. The HA solutions to be used in the experimental studies were freshly prepared by diluting this stock solution with distilled water (5–20 mg/L). pH adjustments of the solutions were performed using NaOH and H_2_SO_4_ from Merck (Darmstadt, Germany). NaOH and KOH solutions were used in desorption studies. In adsorption studies, natural HNTs from the Balıkesir-Çanakkale region, obtained from Esan Eczacıbaşı Industrial Minerals and Raw Materials Inc. (Istanbul, Turkey), were used.

In the filtration experiments, a ceramic UF membrane with a molecular weight cutoff value of 50 kDa, manufactured by TAMI Industries (Nyons, France), was used. The technical specifications of the membrane, which has an active surface coated with zirconium dioxide and a support layer coated with titanium dioxide, are 25 cm in length, and have a 1 cm outer diameter, 0.6 cm inner diameter, and 0.0047 m^2^ active surface area. Experiments were conducted in crossflow mode, and the membranes were stabilized by washing with pure water before each process.

Leachate NF concentrate samples were obtained from the outlet of the NF unit of the leachate treatment plant at the Kömürcüoda Landfill. In order to prevent any chemical or biological reactions, the samples were kept at +4 °C.

### 2.2. Analytical Methods

Parameters such as COD and UV_254_ represent the organic and aromatic contents of the wastewater, which strongly affect membrane fouling. Conductivity and chloride concentration indicate the salinity of the matrix and may influence electrostatic interactions between HAs, HNTs, and the membrane surface. In addition, pH affects the surface charge and adsorption/desorption behavior of both HAs and HNTs. Therefore, characterization of the NF concentrate is essential to evaluate the filtration process and understand membrane performance under real wastewater conditions. In analytical measurements, HA concentrations were determined at a wavelength of 254 nm using a UV-vis spectrophotometer (WTW, Photo Lab 7600). UV_254_ was chosen to represent the presence of aromatic organic compounds and humic-like substances in the samples. Organic compounds that contain aromatic rings and conjugated double bonds typically show strong absorbance near 254 nm. Therefore, UV_254_ measurements were used to estimate the relative amount of aromatic organic matter in the samples [24,25].

The characterization of the NF concentrate was performed in accordance with Standard Methods for the Examination of Water and Wastewater [26]. The pH and conductivity were determined with a WTW Multi 9620 IDS device (WTW, Weilheim, Germany) for pH using the SM 4500-H+-B method, and conductivity was measured according to the SM 2510-B method. The chloride concentration was analyzed utilizing the SM 4500-Cl-B method. Furthermore, color and UV_254_ absorbance were evaluated following the SM 2120-C and SM 5910-B methods, respectively. Finally, the COD was determined using the SM 5220-C method.

Before analysis, all samples were filtered through filters with a pore size of 0.45 µm. Surface morphology and characteristics were evaluated using Field Emission Scanning Electron Microscopy (FE-SEM) via a Thermo Scientific Apreo 2 S LoVac microscope (Thermo Fisher Scientific, Waltham, MA, USA).

Surface chemistry and chemical bond conversions were analyzed via Attenuated Total Reflection-Fourier Transform Infrared (ATR-FTIR) spectroscopy to verify coating success and monitor fouling. Measurements were recorded in the 4000–650 cm^−1^ range using a Shimadzu IRAffinity-1 spectrophotometer (Shimadzu, Kyoto, Japan).

### 2.3. Membrane Coating

A coating process using HNTs was performed to improve the surface properties, increase fouling resistance, and enhance the adsorptive capacity of ceramic UF membranes. The coating process was initiated by homogenizing the HNTs into a suspension in distilled water. Different HNT concentrations (0.05, 0.1, and 0.25 g/L) were tested, and pre-filtration experiments and turbidity measurements determined the optimum coating concentration to be 0.05 g/L.

Under optimum conditions, a 2 L suspension containing 0.05 g/L of HNTs was fed into the tubular ceramic membrane system. The coating process was carried out in crossflow mode at a flow rate of 200 mL/min and a constant pressure of 1 bar for 1 h. During the coating process, turbidity measurements were taken every 15 min, and flux values were recorded. Following the circulation phase, the membranes were dried in an oven at 150 °C for 2 h to ensure stable fixation of the HNT layer on the membrane surface. After drying, the coated membranes were preconditioned by circulating distilled water for 30 min under pressureless conditions. In the final stage, the HNT-coated membranes were first stabilized with distilled water at 1 bar, and the initial pure water flux was recorded. Subsequently, a synthetic HA solution with an initial concentration of 5 mg/L was fed into the system for 2 h at a constant operating pressure of 1 bar and a flow rate of 200 mL/min. To monitor HS retention during filtration, feed and permeate samples were collected every 15 min and analyzed by UV_254_ measurements. After each filtration cycle, the membrane cleaning procedure was performed in two consecutive steps. Initially, the HSs retained on the membrane surface and within the membrane pores were removed by flushing the filtration system. Subsequently, 2 L of 1 M KOH or 1 M NaOH solution, selected based on the batch cleaning experiments, was circulated through the system at an operating pressure of 1 bar and a flow rate of 200 mL/min. Following this alkaline regeneration step, the membranes were removed from the system and sequentially immersed in 0.1 N NaOH at 85 °C for 30 min and 0.04 N H_3_PO_4_ at 50 °C for 15 min. The performance recovery was evaluated based on the final pure water flux obtained after cleaning. It should be noted that this aggressive chemical cleaning step was intended to remove the accumulated organic foulants together with the spent dynamic HNT layer, thereby restoring the ceramic support for the next operation cycle. Therefore, the ceramic support was re-coated with a fresh HNT layer at the start of each subsequent cycle under the same coating conditions. The same coating–filtration–regeneration protocol was subsequently repeated for the next filtration set.

The adhesion of the HNT dynamic layer onto the ceramic support is hydrodynamically driven by the transmembrane pressure and further stabilized by hydrogen bonding between the HNT hydroxyl groups and the membrane’s ZrO_2_ active layer. While permeate flux and retentate turbidity were used strictly as real-time macroscopic indicators to optimize the concentration and ensure sustainable permeability without complete pore clogging, the definitive physical presence, structural homogeneity, and stability of the 0.05 g/L coating layer were subsequently verified using direct FE-SEM characterization. The findings indicated that the 0.05 g/L HNT coating provided effective surface coverage while preserving membrane permeability. The lower turbidity values observed during the coating process, together with the higher pure water flux measured after membrane preparation relative to higher HNT concentrations, indicate that excessive pore blockage was avoided under this coating condition.

### 2.4. Batch Test System for Adsorption and Desorption Studies

Batch adsorption and desorption experiments were conducted to determine the retention and recovery performance of HAs on HNTs. Adsorption studies were carried out with synthetic HA solutions representing leachate NF concentrates with high HA content. To evaluate the effects of key operational variables on the adsorption capacity, experiments were systematically conducted at various initial HA concentrations (2.5–20 mg/L), HNT doses (0.05–1.0 g/L), and temperatures (298, 308, and 328 K). In the standard adsorption stage, suspensions were prepared by adding HNTs at designated concentrations to the HA solution. The mixtures were mixed for 30–45 min at room temperature, at a speed of 180 rpm, and at a pH of 8.54. At the end of the determined contact times, the suspensions were centrifuged at 4000 rpm for 5 min to ensure solid–liquid phase separation. The supernatant was removed and filtered through 0.45 µm filters, and the remaining HA concentration was analyzed. To determine the adsorption capacity, the obtained data were evaluated using the Langmuir and the Freundlich isotherm models and the Lagergren first-order kinetic model. The amount of HAs retained on the HNT surface was calculated according to Equation (1).

(1) $$Retained\;amount\;(mg)=\frac{(C_{0}-C_{t})\times V}{1000}\times 100$$

where C_0_ and C_t_ are the initial and residual HA concentrations (mg/L), respectively, and V is the solution volume (mL). The retained amount represents the mass of HAs adsorbed onto the HNTs at a given contact time.

To better understand the adsorption mechanism, various kinetic models, including pseudo-first-order and pseudo-second-order, were initially evaluated. The pseudo-first-order (Lagergren) model was specifically chosen as it is widely applied for systems involving liquid solutions and effectively describes physisorption processes where diffusion is the rate-limiting step [27]. One of the most commonly used empirical kinetic models, the Lagergren first-order kinetic expression, is defined by Equation (2).

(2) $$\log\left(q_{e}-q_{t}\right)=\log\left(q_{e}\right)-\frac{k_{ad}}{2.303}t$$

where k_ad_ is the first-order rate constant or Lagergren kinetic model rate constant (l/min), and q_t_ is the HA concentration adsorbed at time t (mg/g).

The equilibrium state and thermodynamic parameters of the adsorption process were evaluated using Standard Enthalpy Change (ΔH°), Standard Entropy Change (ΔS°), and Standard Gibbs Free Energy (ΔG°). The enthalpy and entropy values of the adsorption process are determined by Equations (3) and (4):

(3) $$lnK_{d}=\frac{{∆S}^{{}^{\circ}}}{R}-\frac{{∆H}^{{}^{\circ}}}{R\times T}$$

(4) $${∆G}^{{}^{\circ}}={∆H}^{{}^{\circ}}-T{∆S}^{{}^{\circ}}$$

where T is the absolute temperature (K), and R is the ideal gas constant (8.314 J/mol K). The equilibrium constant is defined as the distribution coefficient, K_d_ = Qe/Ce, where Qe is the amount of adsorbed substance per unit adsorbent at equilibrium (mg/g), and Ce is the amount of substance remaining unadsorbed in the solution at equilibrium (mg/L).

The thermodynamic coefficients of the adsorption process were calculated using the graph presented in Figure S3 and summarized in Table S3. The evaluation of these parameters provides significant insight into the adsorption mechanism. The negative standard enthalpy change confirms that the adsorption of HAs onto HNTs is an exothermic process, which is also characteristic of physical adsorption driven by weak intermolecular forces. Furthermore, the negative values of ΔG° indicate that the adsorption of HAs onto the HNT surface is a spontaneous and thermodynamically favorable process. Finally, the ΔS° value reflects the affinity of the adsorbent for the HA molecules and describes the changes in the randomness or degrees of freedom at the solid–liquid interface during the adsorption process.

Desorption studies were carried out with the HA-loaded HNT solid phase obtained at the end of the adsorption step. NaOH and KOH solutions were used as desorbents, and 0.1 M, 0.5 M, and 1.0 M concentrations were tested comparatively for both chemicals. The HA-loaded adsorbent was mixed at room temperature for 60 min by contact with the relevant desorption solution. Intermediate samples were taken at 2, 5, 10, 30, 45, and 60 min to evaluate the desorption process. Following centrifugation and filtration, the concentration of desorbed HAs in the liquid phase was determined spectrophotometrically, and the desorption efficiency was calculated using Equation (5).

(5) $$RR\left(\%\right)=\frac{Amount\;of\;desorbed\;HA\;(mg)}{Amount\;retained\;in\;the\;solid\;phase\;(mg)}\times 100$$

where RR represents the desorption efficiency (%). The amount of desorbed HAs (mg) corresponds to the mass of humic acid released from the HNT surface during the desorption process, whereas the amount of HAs retained in the solid phase (mg) represents the mass of humic acids previously adsorbed onto the HNTs during the adsorption stage. The desorption efficiency reflects the regeneration potential of the adsorbent, with higher (RR) values indicating a greater recovery of adsorbed humic acid and a more effective regeneration of the HNT surface.

### 2.5. Filtration Experiments

**Figure 1:**
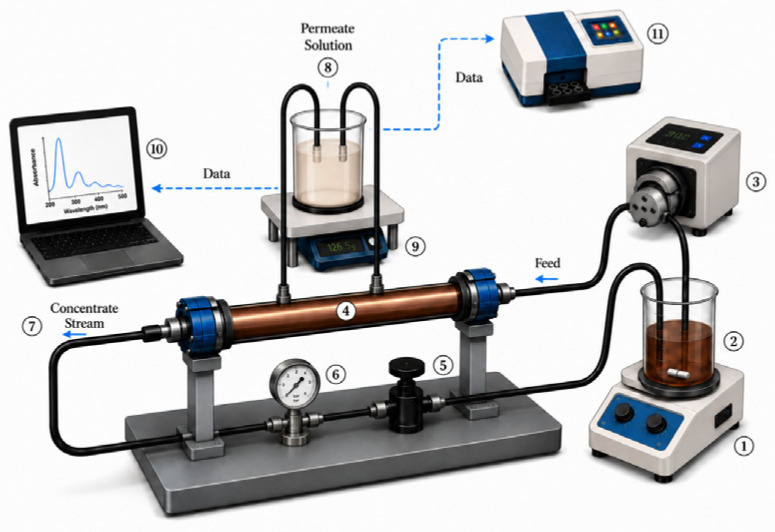
The schematic representation of the experimental procedure (1: magnetic stirrer; 2: feed solution; 3: peristaltic pump; 4: adsorptive ceramic membrane; 5: tubular membrane module; 6: transmembrane pressure valve and gauge; 7: concentrate stream; 8: permeate solution; 9: analytical balance; 10: computer for data collection; 11: spectrophotometer for HA measurement).

The crossflow ceramic membrane system used in the experimental studies consists of a feed tank, peristaltic pump, tubular membrane module, pressure gauge, and data acquisition unit (Figure 1). The system utilizes tubular ceramic UF membranes with a molecular weight cutoff value of 50 kDa, an active surface of zirconium dioxide, and a support layer of titanium dioxide. The filtration process was initiated by placing membranes coated with an optimum HNT concentration of 0.05 g/L, as detailed in Section 2.3. To ensure accurate flux measurements, the HNT-coated membranes were first subjected to a pre-filtration step. Specifically, the membranes were stabilized with distilled water at a crossflow rate of 200 mL/min under 1 bar pressure for 30 min. Following this initial stabilization period, the pure water flux was continuously monitored and recorded for 60 min under the same operational conditions to establish a reliable baseline before proceeding with the experiments. Subsequently, a synthetic HA solution with an initial concentration of 5 mg/L was fed into the system for 2 h under a constant operating pressure of 1 bar and a flow rate of 200 mL/min. To monitor the mechanisms of HA retention on the membrane surface during filtration, samples were taken from the feed and permeate phases every 15 min, and UV_254_ analyses were performed. Following the completion of the filtration phase, desorption experiments were conducted to recover the HSs retained on the membrane surface and in its pores. In this phase, 1 M NaOH and 1 M KOH solutions, which provided the highest efficiency in batch experiments, were used as recovery agents. The prepared 2 L chemical solution was fed into the system at 1 bar pressure and 200 mL/min flow rate, allowing the HAs retained on the membrane to dissolve chemically and pass into the permeate phase. In the final stage of the experiments, the system’s performance in a real wastewater environment was tested with real leachate NF concentrate samples. After the real wastewater experiments, a chemical cleaning procedure was applied to restore the membrane performance. In this context, the membranes were exposed to 0.1 N NaOH at 85 °C (30 min) and 0.04 N H_3_PO_4_ at 50 °C (15 min), respectively. The performance recovery was evaluated based on the final pure water flux obtained after cleaning.

### 2.6. NF Concentrate Characterization

The standard procedures were used when characterizing the NF concentrate sample [26]. UV_254_ was detected to determine the relative amounts of aromatic chemicals in the samples. A UV/Vis spectrophotometer was used to quantify the samples’ UV_254_ measurements both before and after treatment.

### 2.7. Halloysite Nanotube Characterization

A comprehensive characterization of HNTs, specifically evaluating their morphology, specific surface area, and surface functional groups, is fundamental for understanding and optimizing the hybrid separation process. Structurally, the tubular morphology and high specific surface area of HNTs directly dictate the density of accessible active sites, which governs the maximum adsorption capacity for HAs. Chemically, the identification of surface functional groups (e.g., surface hydroxyls) is critical for elucidating the predominant binding mechanisms, such as hydrogen bonding and electrostatic interactions between the HNTs and the target pollutants. Furthermore, from an operational perspective, these intrinsic nanoscale properties heavily influence the structural integrity of the coating; they dictate how the HNT particles interact with the ceramic support, how they pack under crossflow pressure, and ultimately, how they determine the permeability and stability of the dynamically formed secondary layer [28,29,30].

The detailed characterization of the HNTs used in this study has been reported in our previous studies [31,32], and their basic properties are summarized here. The chemical composition of the HNT sample was analyzed by X-ray fluorescence (XRF), and it was determined that the main components forming the basis of the structure are SiO_2_ (44.00%), Al_2_O_3_ (37.59%), and Fe_2_O_3_ (0.69%) [31,32]. The average diameters of the HNTs were reported to vary between 40 and 100 nm, and the length was reported to be around 500 nm [32]. In terms of surface properties, the specific surface area of HNTs was measured as 100.74 m^2^/g by the Brunauer–Emmett–Teller (BET) analysis, and the cation exchange capacity was measured as 24.42 meq/100 g by the methylene blue method [31,32]. The morphology of HNTs was analyzed by Keskin et al. and Ormanci-Acar et al. with a scanning electron microscope and a transmission electron microscope, and it was stated that the distribution is homogeneous and cylindrical [31,32]. According to the TEM results, the cylindrical, hollow, and open-ended structure of the HNTs was confirmed [32].

## 3. Results and Discussion

### 3.1. Batch Adsorption Studies

In studies conducted to determine the adsorption capacity of HNTs, the effects of key variables such as HA concentration, HNT dose, and temperature on HA removal efficiency were investigated. According to the results presented in Figure S1a, the HA removal efficiency tends to decrease as the initial HA concentration increases. The sharp initial increase in the adsorption curves, followed by a flattening trend, is explained by the initial density of vacant spaces on the adsorbent surface and the subsequent filling of these spaces over time, leading to surface saturation. According to the isotherm parameters obtained for HA adsorption presented in Table S1, it was determined that the Freundlich isotherm model fits the adsorption data obtained for all initial concentrations with high R^2^ values. The isotherm and kinetic fitting parameters are presented in Tables S1 and S2, respectively. As shown in Table S1, the Freundlich model provided a better representation of HA adsorption onto HNTs than the Langmuir model, with R^2^ values increasing from 0.894 to 0.947 depending on the initial HA concentration. The Freundlich constants also indicated that HA adsorption occurred on a heterogeneous HNT surface rather than through ideal monolayer adsorption. The kinetic behavior was evaluated using the Lagergren pseudo-first-order model, and the corresponding k_1_ and R^2^ values are summarized in Table S2. The R^2^ values ranged between 0.8788 and 0.9960, indicating an acceptable fit of the experimental data to the pseudo-first-order kinetic model under different HA concentrations, HNT doses, and temperature conditions. These fitting results support the interpretation that HA adsorption onto HNTs is mainly governed by heterogeneous surface interactions and physical adsorption mechanisms. Regarding the effect of adsorbent dosage (Figure S1b), the percentage of HA removal was observed to be a direct function of the HNT dose. As the HNT dose increased, the adsorption rate increased proportionately due to the expansion of the available specific surface area. When evaluating the effect of temperature on the adsorption capacity (Figure S1c), it was determined that the adsorption efficiency decreased with increasing temperature, indicating an exothermic process.

The coefficients of the Lagergren kinetic models applied for different initial HA concentrations (Figure S2a), different HNT doses (Figure S2b), and different temperatures (Figure S2c) are presented in Table S2. Examination of the obtained reaction rate constants revealed that as the initial concentration increased and temperature increased, the rate constants decreased, while as the adsorbent dose increased, the reaction rate constants increased. The variations in the calculated *k_ad_* can be explained by fundamental adsorption kinetics principles. The decrease in *k_ad_* with increasing initial HA concentration is due to the rapid saturation of available active sites; higher concentrations lead to increased competition among HA molecules, making diffusion into the pores the limiting factor and prolonging the time required to reach equilibrium. Furthermore, the decrease in *k_ad_* at elevated temperatures supports the exothermic nature of the system. Higher thermal energy increases the mobility of the adsorbate molecules and weakens the weak intermolecular forces (physisorption) between HA and the HNT surface, thereby lowering the overall adsorption rate. Conversely, the increase in *k_ad_* with a higher adsorbent dose is directly attributed to the expanded total surface area and the greater abundance of available binding sites, which facilitates a faster uptake of HA molecules.

The thermodynamic coefficients of the adsorption process were calculated using the graph presented in Figure S3 and summarized in Table S3.

The calculated negative standard enthalpy change (−12.2 kJ/mol) confirms that the adsorption of HAs onto HNTs is an exothermic process. Furthermore, the magnitude of the enthalpy change provides crucial insights into the governing adsorption mechanism [33]. As shown in Figure S1c, the adsorption efficiency decreased with increasing temperature, indicating an exothermic process. In the literature, the enthalpy change for physisorption is typically reported to be between −20 and 40 kJ/mol [34,35], and physical adsorption heat generally fall in the 5–45 kJ/mol range [36]. In contrast, chemisorption involves much stronger interactions, with enthalpy changes typically ranging from −80 to −400 kJ/mol due to the formation of covalent bonds [35,36,37]. The relatively low magnitude of our ΔH° value falls well within the established thermodynamic range for weak intermolecular forces, carefully confirming that the adsorption of HAs onto HNTs has a physical adsorption character.

### 3.2. Batch Experiments for Desorption

Batch experiments were conducted to evaluate the effect of different alkaline solutions on HA desorption. In all experiments, the amount of adsorbed HAs was calculated as q_ads_ = 9.1 mg/g for 0.5 M and 1 M KOH/NaOH; and q_ads_ = 8.2 mg/g for 1.5 M KOH and 1.5 M NaOH. The amount of recovered HAs (q_des_) and the recovery percentage were evaluated depending on the chemical conditions. Table 1 shows that the recovery efficiency varies depending on the type and concentration of the alkaline solution used. While it was determined that NaOH solution provided a higher desorption efficiency at all concentrations, the difference between 1 M and 1.5 M concentrations is small, and 1 M concentrations appear sufficient for synthetic HA studies to minimize chemical consumption.

### 3.3. Membrane Experiments

#### 3.3.1. HNT Coating Optimization

**Figure 2:**
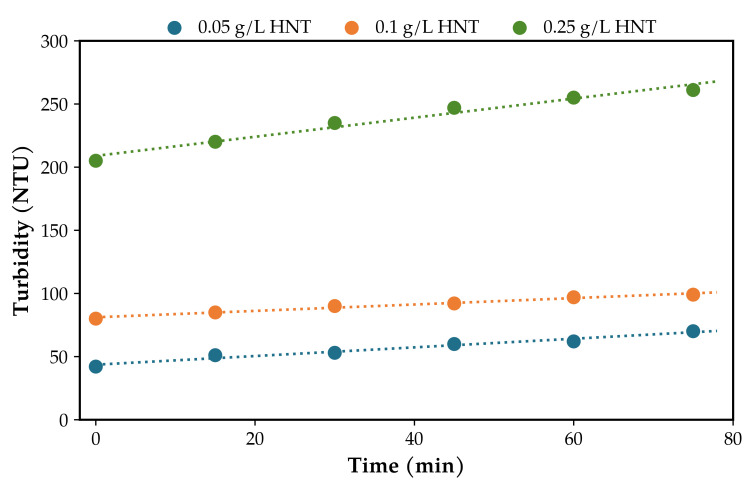
Temporal Variation in Turbidity at Different HNT Concentrations.

To determine the effect of different HNT concentrations on membrane coating performance, coating processes were carried out with feed solutions with concentrations of 0.05, 0.1, and 0.25 g/L. Figure 2 shows the turbidity results obtained in the concentrate stream during the coating process, and it is observed that the turbidity values in the concentrate increase significantly with increasing HNT concentration. The high turbidity values observed, especially at 0.25 g/L, indicate that the HNT concentration is high for the coating and that the coating homogeneity may be negatively affected. In contrast, lower and more controlled turbidity values were obtained at the 0.05 g/L concentration.

**Figure 3:**
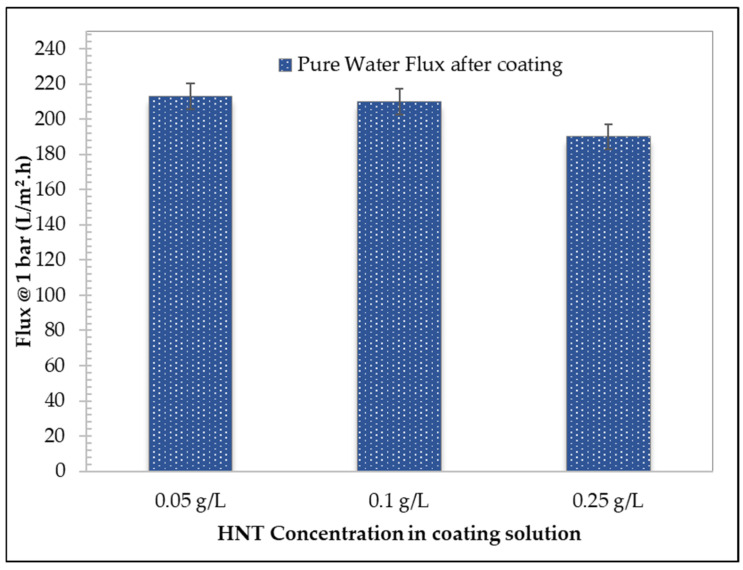
Change in Pure Water Flux of the Membrane After Coating.

After coating, distilled water flux was observed to evaluate the membrane performance (Figure 3). The average values of distilled water equilibrium fluxes for HNT-coated membranes at HNT concentrations of 0.05 g/L, 0.1 g/L, and 0.25 g/L are 216 L/m^2^.h, 212 L/m^2^.h, and 193 L/m^2^.h, respectively. This indicates that increasing the amount of HNTs in the coating solution creates a thick layer on the membrane surface, generating hydraulic resistance to the passage of distilled water. When turbidity and flux results are evaluated together, the optimum HNT coating concentration of 0.05 g/L was chosen due to its low turbidity and high flux values, and the membranes were coated at this value in all experiments.

#### 3.3.2. Membrane Characterization

**Figure 4:**
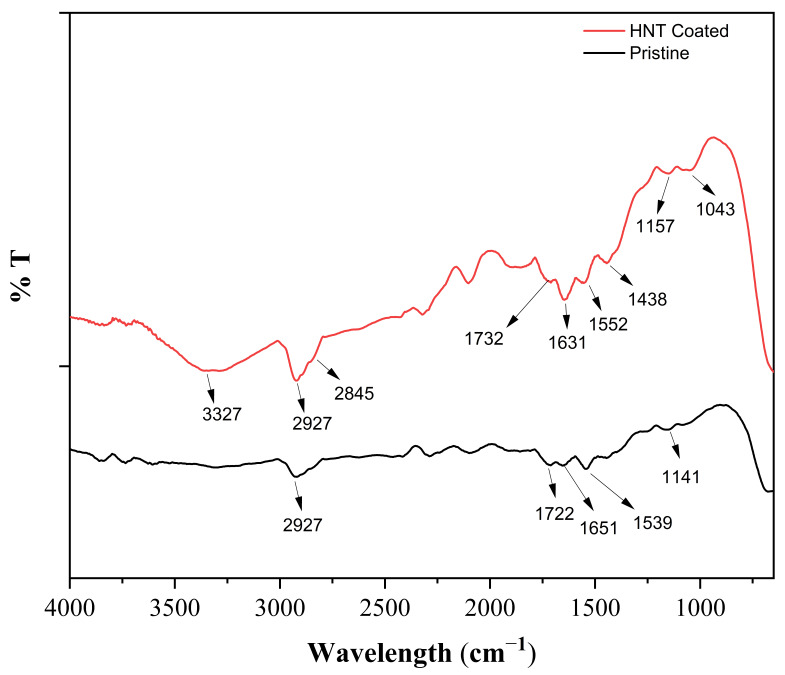
FTIR spectra of the pristine and HNT-coated ceramic membranes.

The surface chemical compositions of the pristine and modified membranes were analyzed using FTIR spectroscopy, as illustrated in Figure 4. The spectrum of the pristine zirconia membrane exhibited typical baseline characteristics, with minor peaks primarily attributed to trace environmental impurities or physically adsorbed moisture (e.g., O-H bending around 1651 cm^−1^). Following the modification, the HNT-coated membrane displayed several new, distinct absorption bands, confirming the successful deposition of halloysite nanotubes onto the zirconia support. The broad and intense band appearing at 3327 cm^−1^ is attributed to the stretching vibrations of the inner-surface and outer hydroxyl (-OH) groups of the HNTs. Furthermore, the pronounced peaks at 1043 cm^−1^ and 1157 cm^−1^ strictly correspond to the characteristic Si-O-Si and Si-O stretching vibrations of the aluminosilicate framework. The peak at 1631 cm^−1^ represents the O-H bending vibration of interlayer water molecules, which is indicative of the highly hydrophilic nature of the HNT layer [38,39]. These significant spectral alterations robustly verify the stable integration of the HNT active layer on the ceramic membrane surface.

**Figure 5:**
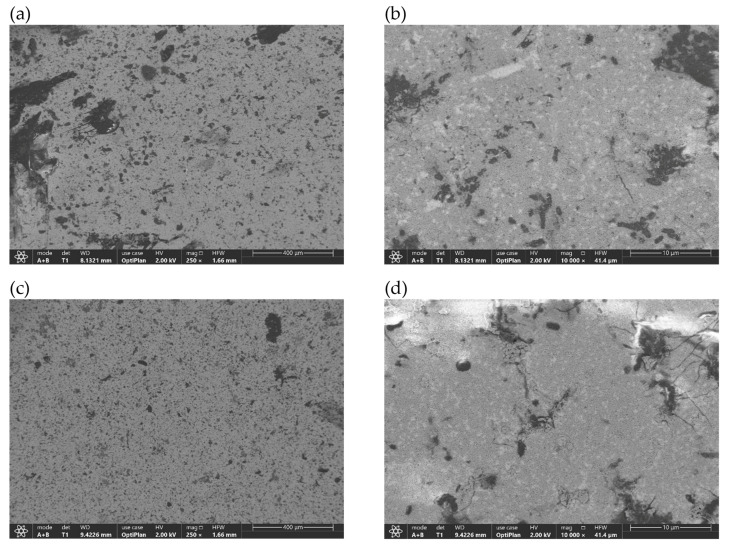
FE-SEM surface micrographs of the ceramic membranes: (**a**) pristine (250×), (**b**) pristine (10,000×), (**c**) HNT-coated (250×), and (**d**) HNT-coated (10,000×).

The surface morphologies of the pristine and HNT-coated ceramic membranes were examined using SEM at different magnifications (Figure 5). The pristine zirconia support (at 250× magnification) exhibits a relatively uniform, crack-free, and flat macroscopic surface profile with inherently distributed microporosity. At higher magnification (10,000×), the sintered granular structure of the zirconia matrix can be observed. This inherent structural roughness and the presence of natural ceramic grains appear to provide a suitable foundation, offering potential anchoring sites for the deposition of the dynamic HNT active layer. Following the coating procedure, morphological alterations are observable in the SEM micrographs (Figure 5). The overall surface view of the coated membrane (250×) suggests that the coating is relatively homogeneously distributed across the macroscopic expanse of the surface. Furthermore, the 10,000× magnification image indicates that the halloysite nanotubes have populated the surface grains and the interstitial voids of the zirconia ceramic. It is observed that the HNT structures tend to form an interconnected, web-like dynamic layer by bridging the surface pores, which implies a stable interaction with the ceramic support. These morphological findings are consistent with the presented FTIR results, further supporting the successful fabrication of the dynamic HNT membrane layer on the zirconia support.

#### 3.3.3. Synthetic HA Experiments

**Figure 6:**
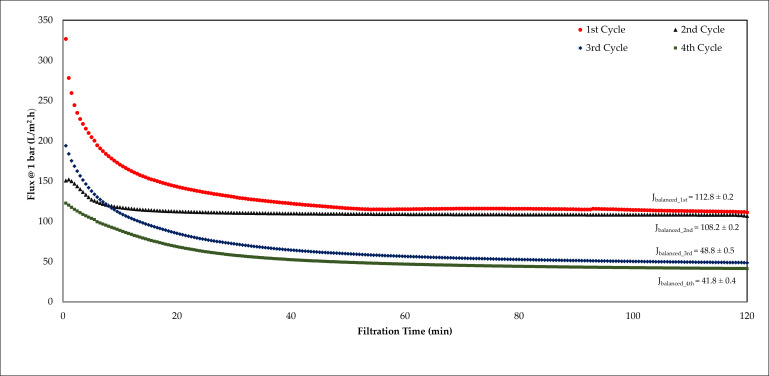
Flux–time profile during HA filtration (HA concentration: 5 mg/L; T: 25 °C; initial pH: 8.44; HNT dose: 0.05 g/L; pressure: 1 bar).

Experiments using a synthetic HA solution were conducted to evaluate the performance of the membrane system under controlled conditions. In this context, the filtration behavior, operational reproducibility, and HA recovery performance of the dynamic HNT-coated ceramic membrane system were investigated through repeated coating–filtration–regeneration cycles. NaOH and KOH were used as regeneration agents. Figure 6 presents the time-dependent flux of the filtration experiments performed at 1 bar pressure and an initial HA concentration of 5 mg/L. Each curve represents a filtration cycle consisting of four steps and showing a two-hour HA flux variation. These four steps include HNT coating, HA filtration, distilled water filtration, and regeneration/chemical cleaning of the membrane. It was observed that the equilibrium flux values decrease as the number of cycles increases. In the first two cycles, the equilibrium flux values were 112.8 ± 0.2 L/m^2^.h and 108.2 ± 0.2 L/m^2^.h, respectively, while in the last two cycles, the flux values decreased to 48.8 ± 0.5 L/m^2^.h and 41.8 ± 0.4 L/m^2^.h. The observed decline in flux across the filtration cycles (from ~112 to ~42 L/m^2^·h) may be attributed to a combination of factors, including the progressive accumulation of foulants on the membrane surface and the potential changes in the membrane’s structural properties. Furthermore, the stabilization of flux values observed in the final two cycles after 10 min suggests that the membrane-coating system has reached a state of hydraulic equilibrium rather than purely indicating the structural stability of the HNT layer.

**Figure 7:**
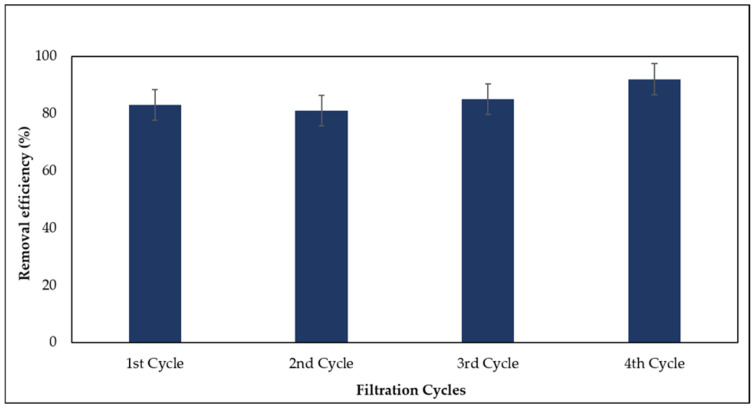
HA removal efficiency over filtration cycles (HA concentration: 5 mg/L; T: 25 °C; initial pH: 8.44; HNT dose: 0.05 g/L; pressure: 1 bar).

Figure 7 presents the HA removal efficiencies obtained during four consecutive membrane filtration runs. In each run, the filtration process was performed for 2 h using the HNT-coated membrane under identical operating conditions. It is observed that the removal efficiency is high in all cycles. While the HA removal efficiencies after 2 h in the first two cycles were 83% and 81%, they reached 85% and 92% in the last two runs. The average removal efficiency was calculated as 85.3 ± 4.2%, demonstrating that the adsorptive separation performance of the HNT-coated membrane remained stable throughout the repeated filtration experiments. The high removal efficiency obtained in the final run indicates the reproducibility of the dynamic HNT coating procedure and the functional consistency of the coating–filtration–regeneration protocol.

HA retention in the HNT-coated ceramic UF membrane system can be associated with multiple simultaneous mechanisms, including HNT-assisted adsorption, surface interactions with the ceramic layer, size-based interception of larger HA fractions or aggregates, and cake-layer-assisted retention during filtration. Although the ceramic UF membrane has a nominal MWCO of 50 kDa, HAs are a heterogeneous and polydisperse organic fraction. Therefore, partial retention by the membrane matrix and the developing fouling layer may also contribute to the overall removal performance. The independent batch adsorption–desorption results and the FTIR/FE-SEM characterization of the coated membrane support the contribution of HNTs to HA retention. A more detailed mechanistic understanding of HA retention in such hybrid systems requires further investigation of the interactions among HA fractions, the ZrO_2_ active layer, HNT surface sites, the dynamically formed coating layer, and the cake/fouling layer developed during filtration. In particular, the effects of HA molecular-size distribution, surface charge interactions, matrix composition, and coating-layer evolution should be considered to clarify the relative contribution of adsorption, surface interaction, and filtration-assisted retention mechanisms.

Operating as a dynamic sacrificial coating layer, the HNT layer is intentionally stripped away along with foulants during harsh chemical cleaning. The sustained HA removal efficiency (up to 92% in the 4th cycle) supports the high reproducibility of the subsequent re-coating process and the chemical resilience of the underlying ceramic support, rather than the survival of a single coating layer.

**Figure 8:**
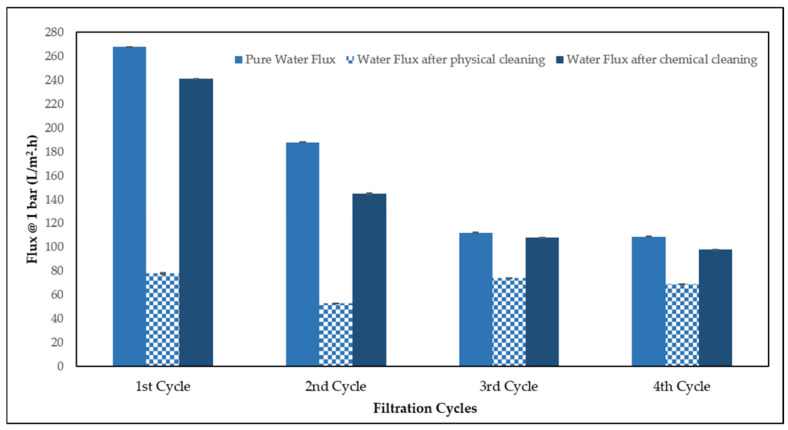
Effect of physical and chemical cleaning on distilled water flux over cycles.

Figure 8 presents the equilibrium flux values of distilled water filtration obtained after coating, physical cleaning, and chemical regeneration in filtration cycles. While the initial fluxes obtained after coating were observed to decrease as the cycles progressed, it was determined that the flux values were only partially recovered with physical cleaning applied after 2 h of filtration with a 5 mg/L synthetic solution. In contrast, it was observed that the flux values increased significantly after chemical cleaning and reached levels closer to the initial fluxes. This indicates that the fouling that occurs can be largely removed by chemical cleaning, but a partially irreversible accumulation occurs as the cycles progress.

Following synthetic HA solution filtration, the membrane was physically cleaned and regeneration processes were performed using 1 M NaOH and 1 M KOH solutions, respectively, after each filtration cycle. In the regeneration study with 1 M NaOH, it was determined that 1.5 mg/L of the organic matter adsorbed from a 5 mg/L HA solution was desorbed, and the desorption efficiency was calculated as 35.9%. Similarly, in the regeneration experiment performed with 1 M KOH, it was determined that 1.6 mg/L of the total retained 4.3 mg/L of organic matter was desorbed, and the desorption efficiency was 36.8%. The results obtained in Table 2 show that both bases showed similar desorption performance; however, the use of KOH provided a slightly higher recovery.

#### 3.3.4. Real NF Concentrate Experiments

In this section of the study, the performance of the system under real wastewater conditions was evaluated based on the findings obtained from experiments conducted with a synthetic HA solution.

Since the real wastewater matrix presents an environment where HSs are present in more complex components, the aim was to investigate the separation and recovery performance of the system under these conditions. In this context, the HA removal and recovery performance, along with the membrane flux behavior, were evaluated in experiments conducted with NF concentrate, and the real application potential of the system was revealed. The characterization of NF concentrate is given in Table 3.

**Figure 9:**
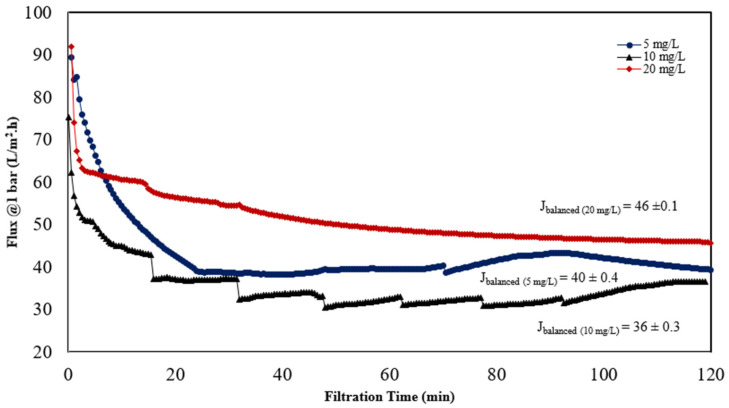
Effect of feed concentration on membrane flux (pressure: 1 bar; T: 25 °C; initial pH values: 7.53, 7.26, and 6.46 for 5, 10, and 20 mg/L, respectively).

Flux change and HA recovery were examined in filtration experiments conducted at 3 different initial concentrations. Figure 9 shows the flux-time changes obtained during the filtration of real NF concentrate samples at concentrations of 5, 10, and 20 mg/L. It was determined that the flux values showed a rapid decrease at the beginning of filtration at all concentrations, followed by a slight decrease until equilibrium was reached. When the concentration effect was examined, it was observed that the initial fluxes changed with increasing concentration, and a significant decrease occurred, especially in the 20 min period. Following this process, the system showed more stable behavior, and equilibrium flux values were reached after approximately 50 min. The equilibrium fluxes obtained at the end of the 120 min filtration period for the real NF concentrate were accurately recorded as 40.0 ± 0.4 L/m^2^.h for the 5 mg/L feed, 36.0 ± 0.3 L/m^2^.h for the 10 mg/L feed, and 46.0 ± 0.1 L/m^2^.h for the 20 mg/L feed concentration, indicating a more complex and matrix-dependent flux behavior compared to the synthetic solutions.

In addition to flux behavior, the NF concentrate filtration results showed that for 5 mg/L NF concentrate, the organic matter removal efficiency was 64.0%, and the adsorption capacity was 40.6 mg/g. For a 10 mg/L NF concentrate, the removal efficiency was calculated as 54.5%, and the adsorption capacity was determined to be 71.7 mg/g. At the highest initial concentration of 20 mg/L NF concentrate, the removal efficiency decreased to 19.0%, and the adsorption capacity was calculated as 44.0 mg/g. The results obtained show that the specific adsorption capacity and the removal efficiency are highly dependent on the initial feed concentration and the complex matrix of the real wastewater.

**Figure 10:**
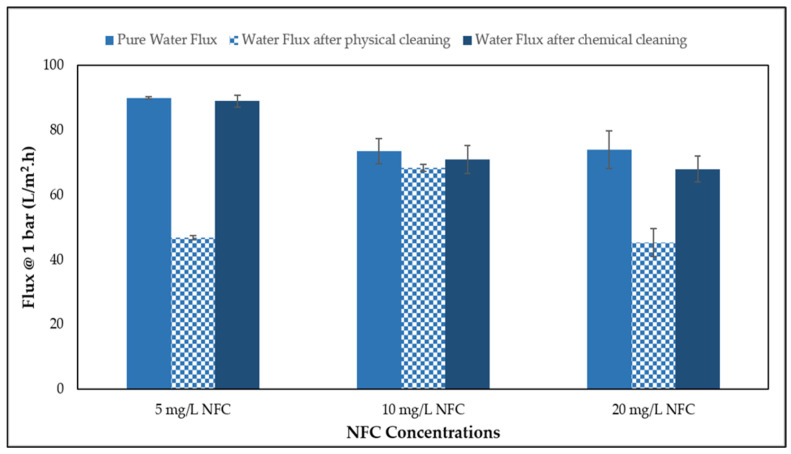
Effect of cleaning methods on flux at different feed concentrations.

After filtration experiments using NF concentrate, membrane cleaning performance was evaluated, and the effects of physical and chemical cleaning steps on flux were investigated. Figure 10 shows the distilled water fluxes measured under 1 bar pressure for three different stages. The first column shows the initial distilled water flux measured after coating, the second column shows the distilled water flux after physical cleaning applied after 2 h of filtration with different feed waters (5 mg/L, 10, and 20 mg/L NF concentrate), and the third column shows the flux obtained after chemical cleaning. When the concentration effect was examined, it was observed that physical cleaning was more effective, especially at 10 mg/L, while the flux values after physical cleaning were lower at 20 mg/L. This indicates that more resistant deposits form on the membrane with increasing concentration.

Table 4 shows the variation in the amount of adsorbed HAs (q_ads_) with different initial concentrations. At concentrations of 5 mg/L, 10 mg/L, and 20 mg/L, the q_ads_ values were determined as 40.6 mg/g, 71.7 mg/g, and 44.0 mg/g, respectively. This non-linear trend indicates that adsorption capacity is governed not only by the initial concentration but also by matrix complexity, surface blocking, and diffusion limitations within the dynamic HNT layer. Similarly, q_des_ reached its highest value at 10 mg/L and decreased markedly at 20 mg/L, suggesting that moderate organic loading provided a favorable balance between adsorption driving force and accessibility of active sites, whereas excessive organic loading promoted macromolecular aggregation and the formation of a dense outer fouling layer. This layer may restrict diffusion into the HNT coating and limit the subsequent release of retained HAs during regeneration. Based on the liquid-phase mass balance of the filtration and regeneration steps, the overall desorption efficiencies were calculated as 57.4%, 59.3%, and 23.5% for the 5, 10, and 20 mg/L concentrations, respectively. The sharp decrease at 20 mg/L further indicates that HAs and humic-like substances were more strongly entrapped within the fouling layer or retained in less accessible regions of the dynamic coating under higher organic loading conditions.

#### 3.3.5. Comparison of Synthetic and Real NF Concentrates

**Figure 11:**
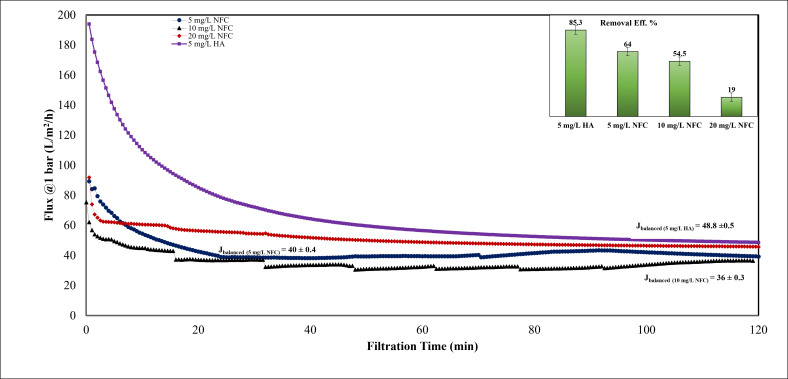
Comparison of the performance between synthetic and real NF concentrates.

The evaluation of the results obtained with the synthetic HA solution and NF concentrate (Figure 11) shows that the system performance varies significantly depending on the matrix structure. Higher removal efficiencies (81–92%) were obtained in the synthetic solution, while these values were significantly lower (19.0–64.0%) for NF concentrate. This sharp decline is attributed to competitive adsorption between HAs and co-existing organic/inorganic species in real wastewater. Similarly, a more stable and predictable flux behavior was observed in the synthetic system, while the flux values were more fluctuating and concentration-dependent in the NF concentrate. In terms of adsorption performance, q_ads_ values increased with increasing initial concentration in the real wastewater (40.6–71.7 mg/g); however, desorption performance did not show a linear increase, and the highest q_des_ value was obtained at the medium concentration (37 mg/g). This non-linear trend suggests that while a moderate increase in concentration provides a stronger driving force into the HNT pores, a higher concentration (20 mg/L) causes rapid macromolecular aggregation on the outer surface. This creates a physical barrier that restricts further diffusion into the inner modified matrix, thereby reducing both adsorption and desorption performance at higher organic loads. This indicates that dissolved organic matter and competitive components in the real wastewater matrix affect the adsorption–desorption balance. Overall, while synthetic solutions offer a suitable model for elucidating the fundamental mechanisms of system performance, the actual wastewater matrix clearly reveals the limitations of the system under real-world application conditions, with lower removal rates, more complex flux behavior, and variable recovery performance.

Nevertheless, the HNT-coated ceramic membrane demonstrated significant advantages over conventional systems. While conventional polymeric membranes frequently suffer from irreversible fouling and require aggressive chemical cleaning when exposed to humic substances [40,41], the HNT-coated ceramic membrane in this study maintained a manageable fouled steady-state. This fouling resistance stems from the hydrophilic nature and tubular morphology of the immobilized HNTs, which reduces the affinity of humic macromolecules toward the surface. Furthermore, the HA removal efficiencies and the relatively rapid establishment of the dynamic layer observed here are highly competitive with other nanoclay-modified membrane systems [23,42]. The ability to successfully recover the adsorbed HAs using simple alkaline desorption (NaOH/KOH) without damaging the structural integrity of the ceramic support highlights a distinct operational advantage over traditional adsorptive membranes, which often exhibit poor reusability [43,44,45]. These comparative findings demonstrate that natural HNTs offer a sustainable, cost-effective, and efficient alternative for modifying ceramic membranes in wastewater recovery applications.

## 4. Conclusions

This study comprehensively evaluated the HA recovery from leachate NF concentrate using an HNT-coated tubular ceramic UF membrane system under both synthetic and real wastewater conditions. The batch adsorption stage showed that HA adsorption onto HNTs was better described by the Freundlich isotherm model, indicating heterogeneous surface adsorption rather than ideal monolayer adsorption. The adsorption process was also highly correlated with the Lagergren pseudo-first-order kinetic model and exhibited an exothermic character, as confirmed by the negative ΔH° value. The optimum HNT dose for membrane coating was determined as 0.05 g/L. The filtration of the synthetic HA solution revealed a high removal efficiency of 85.3% on average throughout the cycles. In experiments conducted with real NF concentrate samples, removal efficiencies remained in the range of 19.0–64.0% because of complex components and competing structures in the wastewater matrix, showing a lower performance compared to the synthetic solution. However, the high desorption efficiency of 59.3% achieved with a 10 mg/L NF concentrate feed proves that the system has significant potential for HA recovery under real application conditions. In conclusion, the combination of HNTs with ceramic membrane technology offers an effective system in the management of leachate NF concentrates and provides the sustainable recovery of HAs.

## Figures and Tables

**Table 1:** Effect of different desorption agents and concentrations on recovery performance.

Desorption Agent	q_ads_ (mg/g)	q_des_ (mg/g)	Desorption Efficiency (%)
0.5 M KOH	9.1	1.9	20.6
1 M KOH	9.1	4.0	41.8
1.5 M KOH	8.2	5.5	67.5
0.5 M NaOH	9.1	4.0	43.26
1 M NaOH	9.1	6.8	73.0
1.5 M NaOH	8.2	6.1	74.6

**Table 2:** HA recovery from HNT-coated membrane (Initial HA concentration: 5 mg/L; HNT dose: 0.05 g/L; T: 25 °C; initial pH (NaOH): 13.8; initial pH (KOH): 14.6).

Chemical	Desorbed HAs (mg/L)	Desorption Efficiency %	q_ads_ (mg/g)	q_des_ (mg/g)
NaOH	1.5	35.9	44.7	30.5
KOH	1.6	36.8	52.5	31.8

**Table 3:** The characterization of NF concentrate.

Parameter	Unit	Value	Method
pH	–	8.45	SM 4500-H+-B
Conductivity	mS/cm	30.3	SM 2510-B
Chloride	mg/L	7898	SM 4500-Cl-B
Color	mg Pt-Co/L	8740	SM 2120-C
UV_254_	1/cm	53.7	SM 5910-B
COD	mg/L	3499	SM 5220-C

**Table 4:** Adsorption and desorption capacities at different feed concentrations.

NF Concentrate	q_ads_ (mg/g)	q_des_ (mg/g)
5 mg/L	40.6	36.8
10 mg/L	71.7	64.6
20 mg/L	44.0	18.0

## Data Availability

The original contributions presented in this study are included in the article/Supplementary Materials. Further inquiries can be directed to the corresponding author.

## Supplementary Materials

The following supporting information can be downloaded at: https://www.mdpi.com/article/10.3390/membranes16070236/s1, Figure S1. Effect of (a) initial HA concentration (T: 25 °C; initial pH: 8.54; HNT dose: 0.5 g/L), (b) HNT dose (T: 25 C; initial pH: 8.54; HA concentration: 5 mg/L), (c) temperature (initial pH: 8.54; HNT dose: 0.5 g/L; HA concentration: 5 mg/L) on HA removal efficiency; Figure S2. Lagergren kinetic models for (a) initial HA concentrations (T: 25 °C; initial pH: 8.54; HNT dose: 0.5 g/L), (b) HNT doses (T: 25 °C; initial pH: 8.54; HA concentration: 5 mg/L), (c) temperature values (initial pH: 8.54; HNT dose: 0.5 g/L; HA concentration: 5 mg/L); Table S1. Isotherm parameters obtained for HA adsorption; Table S2. Lagergren first-order kinetic model coefficients; Figure S3. Thermodynamic graph; Table S3. Thermodynamic coefficients; Figure S4. Experimental design steps.

## Abbreviations

The following abbreviations are used in this manuscript:

BET	Brunauer–Emmett–Teller
DOC	Dissolved Organic Carbon
FA	Fulvic Acid
HA	Humic Acid
HNT	Halloysite Nanotube
HU	Humin
H_2_SO_4_	Sulfuric Acid
HSs	Humic Substances
KOH	Potassium Hydroxide
NaOH	Sodium Hydroxide
NF	Nanofiltration
SEM	Scanning Electron Microscopy
TEM	Transmission Electron Microscopy
UF	Ultrafiltration
XRF	X-ray fluorescence
ΔH°	Standard Enthalpy Change
ΔS°	Standard Entropy Change
ΔG°	Standard Gibbs Free Energy
