# Supplementary material for: Humic Acid Recovery from Leachate Nanofiltration Concentrate Using Halloysite Nanotube-Coated Tubular Ceramic Ultrafiltration Membrane
Source: Membranes (Basel). 2026 Jul 10;16(7):236. doi: 10.3390/membranes16070236 (PMC13413851; doi:10.3390/membranes16070236)
Supplement: Supplementary file 1 [file membranes-16-00236-s001.zip › membranes-4393619-supplementary.pdf]

Supplementary Material

Humic Acid Recovery from Leachate Nanofiltration Concentrate with Halloysite Nanotube-Coated Tubular Ceramic Ultrafiltration Membrane

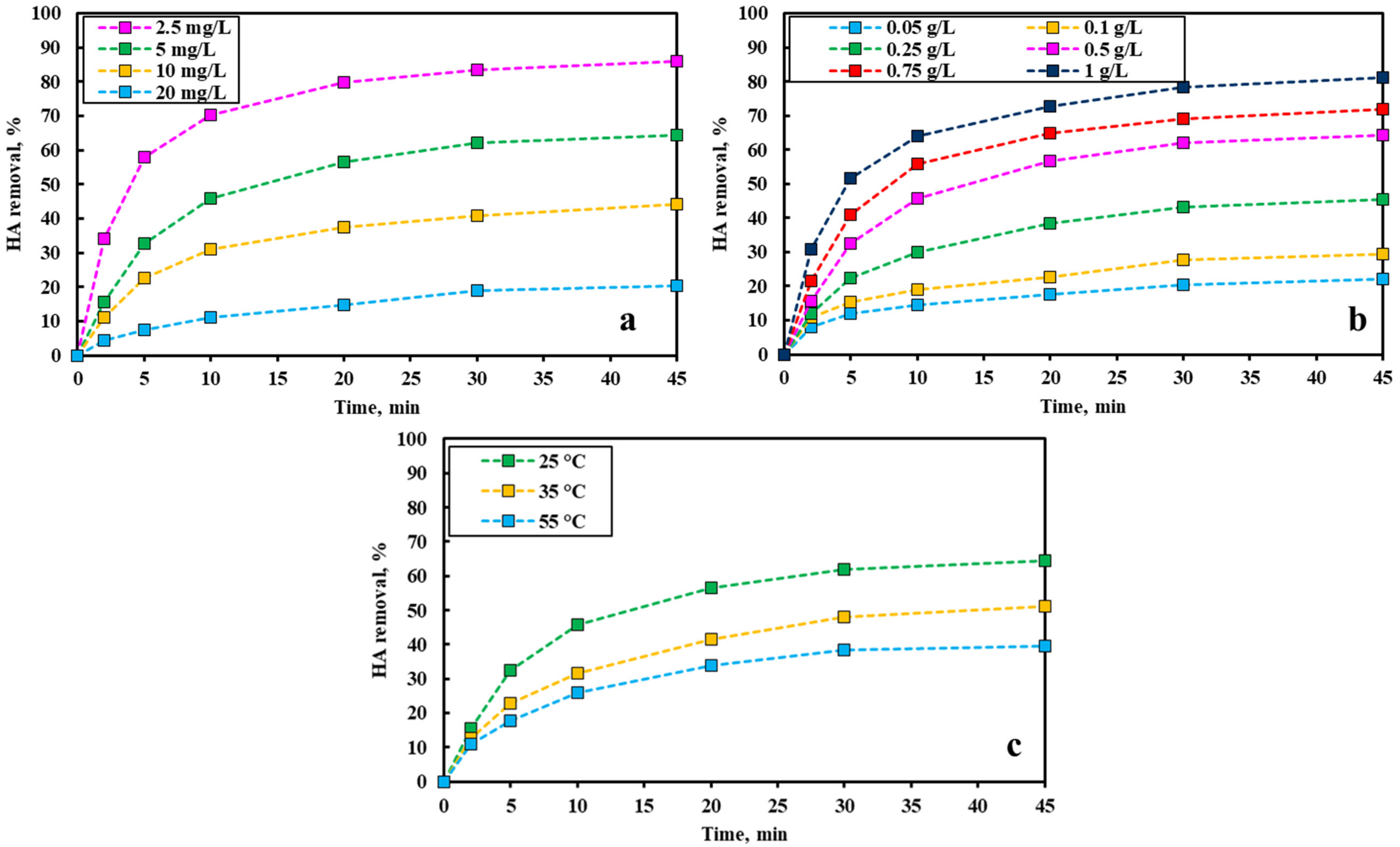

Figure S1. Effect of a) initial HA concentration (T: 25 °C, initial pH: 8.54, HNT: 0.5 g/L) b) HNT dose (T: 25 °C, initial pH: 8.54, HA: 5 mg/L) c) temperature (initial pH: 8.54, HNT: 0.5 g/L, HA: 5 mg/L) on HA removal efficiency.

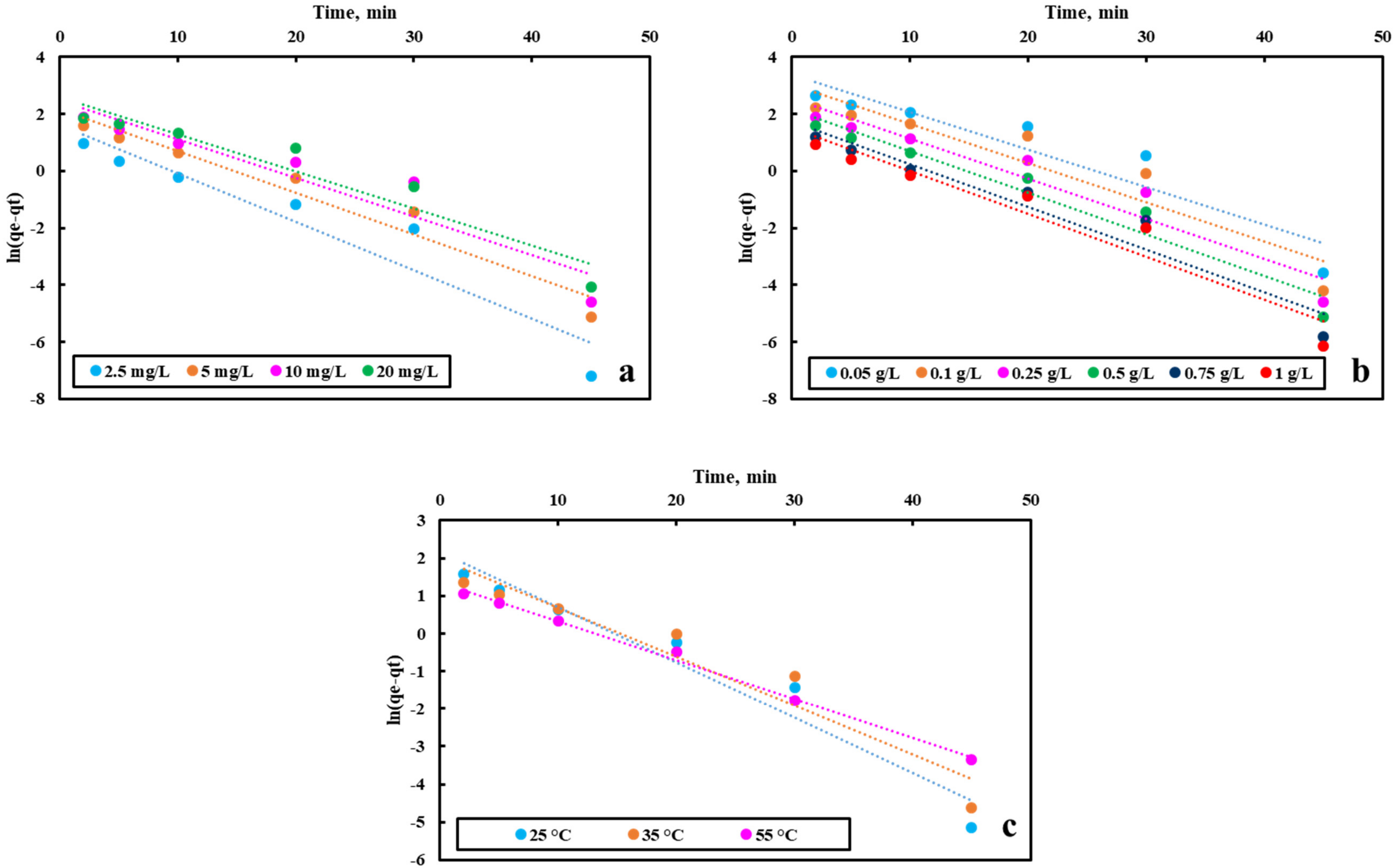

Figure S2. Lagergren kinetic models for a) initial HA concentrations (T: 25 °C, initial pH: 8.54, HNT: 0.5 g/L) b) HNT doses (T: 25 °C, initial pH: 8.54, HA: 5 mg/L) c) temperature values (initial pH: 8.54, HNT dose: 0.5 g/L, HA: 5 mg/L)

Table S1. Isotherm parameters obtained for HA adsorption

	Langmuir parameters			Freundlich parameters		
**HA concentration**	**Q_0 (mg/g)**	**b (L/mg)**	**R^2**	**1/n**	**log K_f**	**R^2**
2.5 mg/L	1.507	3.104	0.944	0.560	0.420	0.894
5 mg/L	1.083	5.787	0.869	1.531	1.245	0.910
10 mg/L	1.029	0.188	0.872	2.871	3.142	0.936
20 mg/L	0.408	0.064	0.837	7.911	10.467	0.947

Table S2. Lagergren first-order kinetic model coefficients

Parameter	Value	k_1 (1/min)	R^2
Initial HA concentration	2.5 mg/L	0.392	0.9051
	5 mg/L	0.337	0.9491
	10 mg/L	0.313	0.8947
	20 mg/L	0.300	0.9109
HNT dose	0.05 g/L	0.304	0.8788
	0.1 g/L	0.318	0.8833
	0.25 g/L	0.324	0.9267
	0.5 g/L	0.337	0.9491
	0.75 g/L	0.346	0.9337
	1 g/L	0.347	0.9273
Temperature (K)	298	0.337	0.9491
	308	0.299	0.9295
	328	0.238	0.996

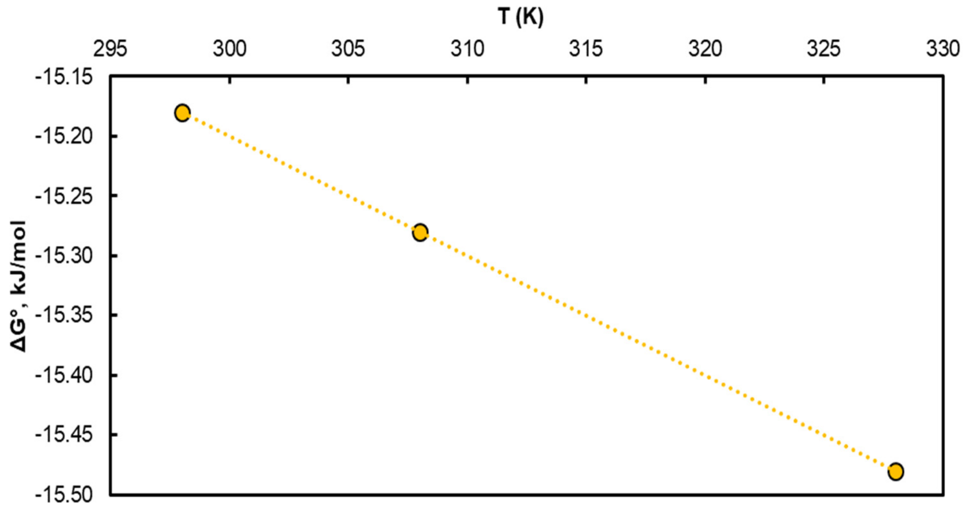

Figure S3. Thermodynamic graph

Table S3. Thermodynamic coefficients

T (K)	1/T	lnKd	ΔG° (kJ/mol)	ΔH° (kJ/mol)	ΔS° (kJ/mol.K)	R^2
298	3.36	6.36	-15.18			
308	3.25	6.07	-15.28	-12.2	0.01	0.9136
328	3.05	5.88	-15.48			

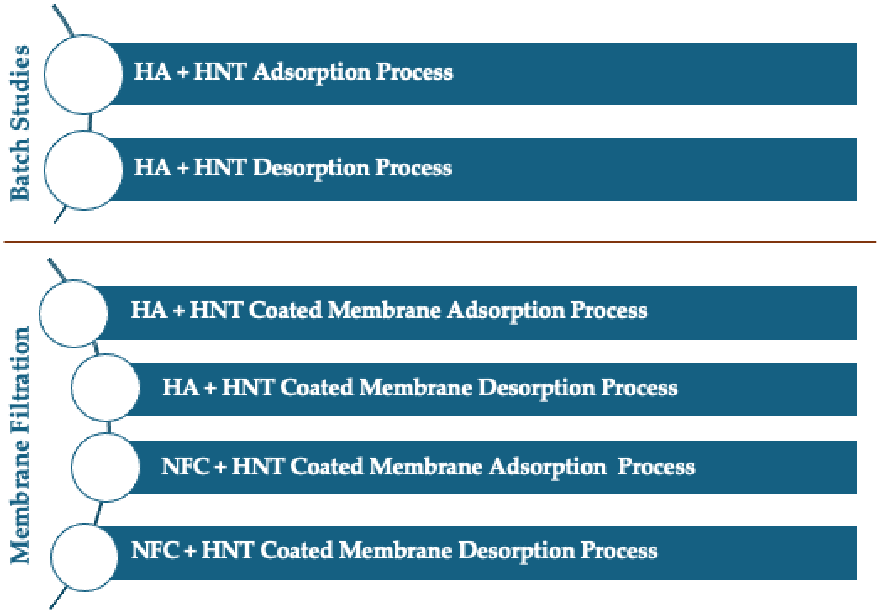

Figure S4. Experimental design steps
